# Risk of Dementia in The Patients with Inflammatory Arthritis

**DOI:** 10.1002/alz70860_102392

**Published:** 2025-12-23

**Authors:** Jun Hong Lee

**Affiliations:** ^1^ National Health Insurance Service Ilsan Hospital, Goyang‐si, Gyeonggi‐do, Korea, Republic of (South)

## Abstract

**Background:**

Inflammation is an important mechanism of dementia. Inflammatory cells and cytokines largely contribute the pathophysiology of inflammatory arthritis. We evaluated the risk of dementia between the patients of inflammatory arthritis and matched population, using data from National Health Insurance Service.

**Method:**

We defined the patients of ankylosing spondylitis, seropositive rheumatoid arthritis, and psoriatic arthritis and enteropathic spondyloarthropathy, using the combination of main diagnosis and V code of rare incurable diseases. Control group was defined by 1:5 propensity score matching in each disease. Newly developed dementia was defined as the patients with the main diagnosis of (G300, G301, G308, G309, F000, F001, F002, F009) and prescription of dementia medication.

**Result:**

In a multivariate analysis including dementia risk factors (hypertension, diabetes, dyslipidemia, and depression), a diagnosis of rheumatoid arthritis with a positive serologic test was associated with a greater incidence of dementia with HR 1.10, 95% CI 1.02‐1.20 (*p* = 0.0159)., the incidence of dementia was lower in the group that used biological agents than in the group that did not (HR 0.46, 95% CI 00.33‐0.65, *p* <0.0001). Among biological agents, the group using non‐TNF blocker had a significantly lower incidence of dementia (HR 0.37, 95% CI 0.21‐0.65, *p* = 0.0005).

**Conclusion:**

In the case of rheumatoid arthritis with a positive serological test, this study also showed a high risk of developing dementia, similar to the results of other previous studies. Regarding changes according to the administration of biological agents, previous studies have shown that anti‐TNF antagonists lower the risk of developing dementia, but this study also showed that the administration of biological agents lowers the risk of developing dementia, and anti‐TNF antagonists Rather, in the case of non‐anti‐TNF antagonists, a statistically significant decrease was shown.

